# Full-Exon Resequencing Reveals Toll-Like Receptor Variants Contribute to Human Susceptibility to Tuberculosis Disease

**DOI:** 10.1371/journal.pone.0001318

**Published:** 2007-12-19

**Authors:** Xin Ma, Yuhua Liu, Brian B. Gowen, Edward A. Graviss, Andrew G. Clark, James M. Musser

**Affiliations:** 1 Center for Human Bacterial Pathogenesis, Department of Pathology, Baylor College of Medicine, Houston, Texas, United States of America; 2 Laboratory of Human Bacterial Pathogenesis, Rocky Mountain Laboratories, National Institute of Allergy and Infectious Diseases, National Institutes of Health, Hamilton, Montana, United States of America; 3 Department of Molecular Biology and Genetics, Cornell University, Ithaca, New York, United States of America; 4 Center for Human Molecular and Translational Infectious Diseases Research, The Methodist Hospital Research Institute, Houston, Texas, United States of America; University College London, United Kingdom

## Abstract

Tuberculosis (TB) is the leading cause of death worldwide due to an infectious agent. Data have accumulated over decades suggesting that variability in human susceptibility to TB disease has a genetic component. Toll-like receptors (TLRs) play a critical role in initiating the innate immune response to many pathogens in mouse models, but little is known about their role in human infections. Human TLRs have been reported to recognize mycobacterial antigens and initiate an immune response. We tested the hypothesis that amino acid-altering polymorphisms in five TLRs were associated with susceptibility to TB disease using a population-based case-control study with 1,312 adult TB patients and controls. Full-coding region sequencing of the five TLR genes in all 1,312 subjects yielded a data set in excess of 16 Mb. Rare nonsynonymous polymorphisms in *TLR6-TLR1-TLR10* were significantly overrepresented among African-American TB cases compared with ethnically-matched control subjects. Common nonsynonymous polymorphisms in *TLR6-TLR1-TLR10* also were significantly associated with TB disease in certain ethnic groups. Among African Americans, homozygotes for the common-variant haplotype TLR1-248S, TLR1-602I, and TLR6-249S had a significantly increased TB disease risk. A transmission/disequilibrium test on an independent sample found that the TLR1-248S variant was preferentially transmitted to diseased children, thereby confirming disease association. These results are consistent with recent reports implicating TLR1 variants, including TLR1-602, in significantly altered innate immune responses. Also consistent with disease association, rare TLR6 variants were defective in their ability to mediate NF-κB signal transduction in transfected human cells. Taken together, the data suggest that variant TLRs contribute to human susceptibility to TB disease. Extensive full-exon resequencing was critical for revealing new information about the role of TLRs in human-pathogen interactions and the genetic basis of innate immune function.

## Introduction

Tuberculosis (TB), an infectious disease caused by *Mycobacterium tuberculosis* (MTB), is a leading public health problem in the United States and throughout the world [1]. Development of active TB disease depends on a complex relationship between the bacterium and the host. Importantly, only about 10% of presumed immunologically normal individuals who are exposed to MTB will develop disease in their lifetime. The molecular mechanisms responsible for susceptibility to clinical disease caused by this pathogen are poorly understood.

Many lines of evidence suggest that host genetics contributes to the susceptibility of humans to developing disease after exposure to MTB [2]–[6]. Familial occurrence, and higher rates of TB disease concordance among monozygotic than dizygotic twins, have been reported [2], [3]. In addition, studies have identified associations between polymorphisms in several candidate genes and clinical TB [4]–[14]. For example, alleles of genes encoding HLA class II antigens, mannose-binding protein, natural resistance-associated macrophage protein 1 (NRAMP1), vitamin D-receptor, interleukin-1 (IL-1), IL-1 receptor, IL-8, IL-12 receptor, MCP-1, DC-SIGN, SP110, TIRAP, MAL, and interferon-γ have been reported to be associated with TB disease in some populations [4]–[14]. However, with few exceptions, the disease-associated alleles reported in these studies have only relatively modest effects, suggesting that additional susceptibility genes remain to be discovered.

Toll-like receptors (TLRs) are a family of proteins that play a key role in the innate immune response to infectious agents through their ability to discriminate conserved microbial structures, known as pathogen-associated molecular patterns (PAMPs), from self [15]. TLR recognition of PAMPs such as lipopolysaccharide (LPS), teichoic acid, and surface lipoproteins, initiates signal transduction through the NF-κB pathway [15]. Nuclear translocation of NF-κB induces transcription of proinflammatory cytokine genes essential to mounting a protective immune response and host defense. Different TLRs recognize distinct classes of products synthesized by pathogens. For example, TLR4 is a receptor for LPS made by Gram-negative bacteria [16]. Consistent with this role, Arbour *et al*. [17] reported that amino acid polymorphisms in the extracellular domain of TLR4 conferred altered responsiveness to inhaled LPS in humans. Among the human TLR family members described to date, TLR1, TLR2, TLR3, TLR4, TLR5, TLR6, and TLR9 have been implicated in the recognition of bacterial components [15]. Associations between TLR polymorphisms and susceptibility to infectious agents have been reported [18]–[21]. However, the conclusions that can be drawn from these studies are limited because, in general, relatively few polymorphic sites have been analyzed, small patient populations have been studied, and the analyses have largely been confined to a single *TLR* gene.

TLRs have been reported to participate in the interaction of pathogenic mycobacteria or their extracellular products with mice and humans [22]–[34]. In particular, TLR2 in association with TLR1 and TLR6, and TLR4 have been implicated as receptors involved in the recognition of mycobacterial antigens and activation of macrophages and dendritic cells (DCs) [28]–[35]. However, with few exceptions [36], [37] there has been little investigation in human patients of the role of *TLR* polymorphisms in susceptibility to TB disease, reflecting a general lack of understanding of this topic in human infections. Here we used full-exon sequencing of *TLR1*, *TLR2*, *TLR4*, *TLR6*, and *TLR10* to test the hypothesis that TLR variants are significantly associated with TB disease in humans.

## Results

### Overview of Sequence Variation in the *TLR*s

**Figure 1:**
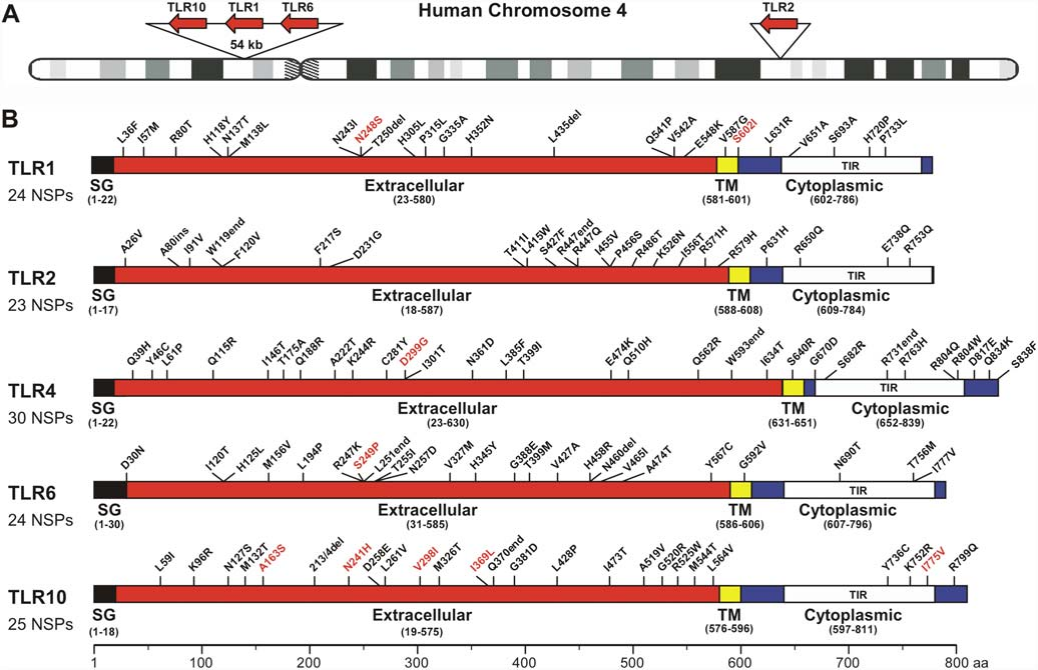
Schematic of the TLR proteins and sequence variants. (*A*) Schematic of human chromosome 4 showing location of TLR genes. (*B*) Summary of variants identified in five TLR proteins. Shown are all variant sites that would be made by the *TLR* alleles identified in either the TB patients or control subjects. Black, rare variant; red, variants that are common in all three ethnic groups analyzed. SG, signal peptide; TM, transmembrane domain; TIR, Toll/IL-1 receptor domain; NSP, nonsynonymous polymorphisms (includes nonsynonymous single nucleotide polymorphisms, nonsense mutations, and insertion and deletions). Numbers in parentheses denote amino acid residue positions.

Analysis of sequence variation in the coding regions of the five *TLR*s in the 1,312 subjects from three ethnicities (African American, European American, and Hispanic) identified 179 sequence variants including 126 nonsynonymous polymorphisms (NSP, altering the primary amino acid sequence) and 53 synonymous single nucleotide polymorphisms (sSNPs, not resulting in an amino acid replacement) (Figure 1B and Table S1). On average, each *TLR* had 25 NSPs (range 23–30 variants), and 10 sSNPs (range 7–18). Thus, NSPs were more abundant than sSNPs by a ratio of 2.5:1, a larger number than is typically observed [38]. Most polymorphic sites were single nucleotide polymorphisms (SNP; 174/179 variants), reflecting lower than average indel polymorphism [38]. Transition substitutions were more prevalent (130/174; 74.7%) than transversions (44/174; 25.3%) among these SNPs, a result consistent with other studies of human DNA sequence variation [38]. Among the nonsynonymous differences are included four rare deletions (1–5 bp in length), one insertion (1-bp), and six nonsense variants (SNPs generating stop codon) (Figure 1B and Table S1).

The proportion of amino acid residues that have a nonsynonymous polymorphism among the five target genes was very similar, being 3.1% in *TLR1* (24/786 amino acids), 2.9% in *TLR2* (23/784), 3.6% in *TLR4* (30/839), 3.0% in *TLR6* (24/796), and 3.1% in *TLR10* (25/811). Twenty-four of the NSPs were present in all three ethnic groups, 22 in two ethnic groups, and 80 NSPs were found in only one ethnic group, of which 53 NSPs presented only once in one single individual (Figure 1B and Table S1). No significant variation was observed among the three ethnic groups in the frequency of occurrence of unique NSP per individual (Table 1).

**Table 1:** Distribution of common and rare nonsynonymous variants of human *TLR* genes among different ethnic groups.

Ethnicities	*TLR1*	*TLR2*	*TLR4*	*TLR6*	*TLR10*	Total
African American (*n* = 533)	5/13*	0/17	2/16	5/13	5/14	17/73
European American (*n* = 290)	3/7	0/7	2/8	1/7	6/6	12/35
Hispanic (*n* = 489)	2/9	0/6	1/12	2/12	6/14	11/53
Total unique NSPs	24	23	30	24	25	126

* Number show the number of nonsynonymous common/rare variants in the target genes based on a cutoff line of allelic frequency 0.03 for common and rare variants in each single ethnicity. NSPs, nonsynonymous polymorphisms.

Four of the five *TLR*s we studied (*TLR1*, *TLR2*, *TLR6*, and *TLR10*) are encoded by chromosome 4, and *TLR6-TLR1-TLR10* comprises a gene cluster located within a 54-kb region of 4p14 (Figure 1A). TLR1 and TLR6 share 69% amino acid sequence identity, TLR1 and TLR10 have 49% sequence identity, and TLR6 and TLR10 have 47% sequence identity. In contrast, TLR2 and TLR4 have much lower identity with each other and other TLRs (range, 20%–30%). Alignment of TLR1, TLR6, and TLR10 revealed that 20 of 73 (27.4%) NSPs were located at amino acid sites conserved among these TLRs. This result may indicate the functional importance of these rare or common NSPs. There were 39 (53.4%) NSPs at homologous residues in more than one TLR, a figure that exceeds the number expected by chance. This result suggests that intergenic gene conversion may be contributing to the variability seen in the *TLR6-TLR1-TLR10* cluster.

### Genotype Distribution

All polymorphic nucleotide sites present in the five *TLR*s were bi-allelic. Treating each polymorphic site as a locus with two alleles, we tested the hypothesis that the control subjects were taken from a population in Hardy-Weinberg equilibrium. Of the 591 single SNP tests for Hardy-Weinberg proportions, 17 had a *P* value less that 0.05, a figure somewhat less than expected based on chance alone. After correcting for multiple comparisons, there was no significant deviation toward an excess or deficit of expected heterozygosity (data not shown).

### Rare NSPs in the *TLR6-TLR1-TLR10* Gene Cluster Are Significantly Overrepresented Among African American TB Cases

We tested the hypothesis that TB patients and control subjects differed significantly in the frequency of NSPs in the *TLR6-TLR1-TLR10* gene cluster. For this analysis, NSPs were divided into two groups, designated as common (allele frequency≥3%) and rare (allele frequency<3%) NSPs. Inasmuch as polymorphisms were present at different allelic frequencies in the three ethnic groups studied, we defined each variant as rare or common based on its frequency in each ethnic group (including cases and controls) (Table 1). Due to the low allele frequencies of rare variants, and findings reported for TLR4 in patients with meningococcal infection [18], we pooled all rare NSPs within each *TLR* and compared the frequencies of rare alleles between cases and controls in each ethnic group (Table 2). Compared to control subjects, African American tuberculosis cases had a significantly increased frequency of rare NSPs in *TLR1* (OR = 3.32; *P* = 0.002), *TLR6* (OR = 1.85; *P* = 0.040), and *TLR10* (OR = 1.76; *P* = 0.009) (Table 2). In contrast, European American cases had fewer rare NSPs in these genes (ORs<1.00) compared to the controls, but only showed statistical significance in *TLR10* (OR = 0.34; *P* = 0.006). In addition, *TLR2* had significantly more rare NSPs among Hispanic controls than the cases (OR = 0.28; *P*<0.001). There was no significant association found between rare NSPs in *TLR4* and TB disease across three ethnic groups, a result consistent with a previous study of *TLR4* SNPs [39]. Taken together, the results support the idea that rare NSPs in several *TLR*s are associated with altered susceptibility to TB disease. In no case was there a significant association between the count of rare synonymous SNPs and disease status.

**Table 2:** Association between rare NSPs of *TLR1, 2, 4, 6, 10* and TB disease

	African Americans		European Americans		Hispanics	
Genes	Cases *n* = 339	Controls *n* = 194	Cases *n* = 180	Controls *n* = 110	Cases *n* = 375	Controls *n* = 114
***TLR1***						
Rare NSPs*	12	5	4	4	9	5
Rare alleles†	39	7	9	9	41	13
OR, (*P* value)‡	3.32	(*0.002*)	0.60	(*0.284*)	0.96	(*0.892*)
***TLR2***						
Rare NSPs	15	5	5	3	6	4
Rare alleles	27	11	13	7	15	15
OR, (*P* value)	1.42	(*0.331*)	1.14	(*0.783*)	0.28	(<*0.001*)
***TLR4***						
Rare NSPs	13	8	4	4	10	4
Rare alleles	42	33	4	5	30	13
OR, (*P* value)	0.71	(*0.156*)	0.48	(*0.272*)	0.69	(*0.272*)
***TLR6***						
Rare NSPs	11	5	6	4	11	3
Rare alleles	47	15	12	8	19	8
OR, (*P* value)	1.85	(*0.040*)	0.91	(*0.846*)	0.71	(*0.431*)
***TLR10***						
Rare NSPs	13	9	3	6	14	5
Rare alleles	90	31	10	17	46	9
OR, (*P* value)	1.76	(*0.009*)	0.34	(*0.006*)	1.59	(*0.210*)

* Number of nonsynonymous polymorphisms (NSPs) identified in each group.

† Number of alleles carrying rare nonsynonymous variants.

‡ Odds ratio (OR) and *P* value were calculated by comparing the numbers of alleles with rare nonsynonymous variants between the cases and controls in each ethnic group.

### Association of Common NSPs in the *TLR6-TLR1-TLR10* Gene Cluster with TB Disease

Nine NSPs were common in all three ethnic groups (Table S2). *TLR2* lacked common NSP variants. Genotypic frequencies for all 21 common NSP were compared between cases and controls within corresponding ethnic groups. None of the three common NSPs in *TLR4* was associated with TB disease. In contrast, 15 of the 18 common NSPs in the *TLR6*-*TLR1*-*TLR10* gene cluster were significantly associated with TB disease in one or more of the ethnic groups (ORs from 1.63–6.77) (Table S2). This result provided evidence that NSPs in the *TLR6*-*TLR1*-*TLR10* gene cluster contributed to human susceptibility to TB disease.

Two common *TLR1* NSPs (N248S and S602I) were in linkage disequilibrium and had reciprocal allele frequencies of minor and major alleles between African Americans and European Americans (Table S2). For example, the G and A alleles of N248S had allele frequencies of 0.789 and 0.211 respectively, in African Americans compared with 0.116 and 0.884, respectively in European Americans. Alleles of both NSPs (248S and 602I) were associated with TB disease in African American patients. N248 (common in European Americans) is a conserved amino acid site in the extracellular domain of TLR1 and TLR6 (N253), and is a putative glycosylation site. Replacement of the Asn residue with Ser might result in altered glycosylation, potentially changing TLR1 folding or function (e.g., PAMP recognition or signal transduction). The S602I amino acid replacement alters the first amino acid of the inferred intracellular domain of TLR1, and this residue varies in TLR10 (L602) and TLR6 (I607). The odds ratios for the tests of association of TLR1 N248S and S602I with TB in African Americans were 1.63 and 2.50 (with tail *P* values of 0.009 and <0.001 respectively), whereas the respective odds ratios in European Americans were 0.20 and 0.49 (neither significant). The two-locus analysis for these SNPs however yielded odds ratios for the highest-risk genotype of 2.68 and 1.75 in African Americans and European Americans, with corresponding *P*-values of <0.001 and 0.007. The two-SNP haplotype contributed more to enhanced susceptibility to TB disease than did the single SNPs in our sample. These results are consistent with recent reports [21], [31]–[34] implicating TLR1 common variants such as S248N and S602I, and rare variants H305L and P315L in altered innate immune responses to lipopeptides and extracts of *M. tuberculosis*, and differential susceptibility to leprosy.

### Functional Analysis of Rare *TLR6* Variants Associated with TB

The genetic epidemiology data and the population genetic analysis suggested that, as a population, rare TLR variants contribute to human susceptibility to TB disease. If this is the case, it is reasonable to expect that some of the rare TLR variants have altered function. Because it was not practical to study the functional aspects of all TLR variants, we elected to focus our efforts on several TLR6 variants. This TLR was chosen for analysis because it has been implicated in MTB-host interaction in the mouse, and reporter assays were available at the time when the nucleotide sequence data were completed. It has previously been shown with Chinese hamster ovary (CHO) cells that mouse TLR6 and TLR2 associate in a ligand-independent manner to form a functional complex that constitutively signals through the NF-κB pathway [40]. Similar studies conducted with HEK 293 cells demonstrated NF-κB activation as a consequence of the co-expression of human TLR2 and TLR6, and found that the cytoplasmic domains of these TLRs were necessary for signaling [41].

**Figure 2:**
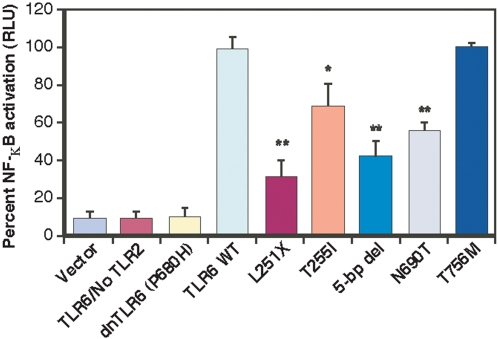
Altered NF-κB signal transduction by rare variants of TLR6. HEK 293 cells were transiently co-transfected with *TLR2* and the indicated *TLR6* variant expression plasmids, NF-κB reporter (firefly luciferase), and β-actin *Renilla* luciferase plasmids. NF-κB activation in transfected cells was measured 24 hrs post transfection. The data shown are the mean ± SD from 4 independent experiments expressed as the percent relative firefly luciferase activity (RLU) (normalized to *Renilla* luciferase activity) induced in cells co-expressing *TLR2* and WT *TLR6*. * *P*<0.01; ** *P*<0.001 compared to TLR6 WT NF-κB activity.

Thus, we hypothesized that the TLR6 proteins resulting from rare mutations that truncate TLR6, or mutations that alter the Toll/interleukin-1 receptor (TIR) domain by a single amino acid, would be defective in signal transduction. To test this hypothesis, we generated TLR6 cDNA vectors expressing wild-type TLR6, a dominant negative form of TLR6 (Pro680His), and 5 TLR6 variants. Of the 5 rare variants examined, 2 were truncated receptors, the other two each had a single amino acid substitution in the TIR domain. The *TLR6* constructs were co-transfected with a human *TLR2* expression vector and an NF-κB reporter into HEK 293 cells and their ability to mediate NF-κB activation was assessed. As predicted, the Pro680His dominant negative replacement completely abolished NF-κB activation (Figure 2). The truncated TLR6 variants (Leu251stop and codon 460/461-5 bp deletion) and one of the TIR domain variants (Asn690Thr) were significantly impaired in their ability to induce NF-κB activation compared to wild-type TLR6 (Figure 2). The rare variant (Thr255Ile) characterized by a substitution in a region of the extracellular domain containing a cluster of rare polymorphisms (Figure 1), also had a significant decrease in NF-κB signaling (Figure 2).

TaqMan real-time RT-PCR analysis was used to verify that there was no significant difference in the transcript level from the various TLR6 constructs, indicating that the observed deficiencies in signaling were not due to differences in the level of gene expression from the constructs used (data not shown). Of note, Thr756 is one of two amino acids in the entire TIR region that varies between humans and chimpanzees, whereas the Asn690Thr replacement is located in a region of the molecule that is well conserved in human, chimpanzee, and mouse TLR6 (data not shown). These observations suggested that an amino acid replacement at Asn690 would have a more substantial detrimental effect on NF-κB signaling than variation located at Thr756. This prediction was borne out by the results of our functional studies (Figure 2).

### Linkage between the TLR1-248S Variant and TB Disease in Children

To further assess the finding from the adult case-control study that TLR1 N248S and S602I were associated with TB disease susceptibility, a separate family-based study was conducted to evaluate the linkage between these two common variants and TB disease. Among the 99 families studied, 64 families with both parents available were informative for conventional transmission/disequilibrium test (TDT) analysis by having at least one parent who was heterozygous for N248S. A total of 99 transmissions of the A (Asn) or G (Ser) allele of the TLR1 248 codon occurred from heterozygous parents to affected children. The G allele was transmitted 61 times compared to 38 times for the A allele, a significant excess (*P* = 0.021) (Table 3). A total of 53 families with heterozygous parents of S602I were informative. We observed 83 transmissions of S602I T or G allele in these 53 families. The T allele was transmitted 44 times (53.0%) compared with 39 times of the G allele (47.0%) (*P* = 0.875). The results provided additional evidence supporting the association between N248S and increased TB disease. The lack of linkage between S602I and TB disease in this TDT study suggested that this variant might be less important in pediatric than adult TB susceptibility.

**Table 3:** TDT analysis of N248S and S602I in families with pediatric TB cases

	Transmitted	Non-Transmitted	χ^2^	*P*
G allele of N248S	61 (61.6%)	38 (38.4%)	5.34	0.021
T allele of S602I	44 (53.0%)	39 (47.0%)	0.30	0.875

## Discussion

### TLR Variants and Susceptibility to TB Disease

Several lines of evidence suggest that TLR variants confer enhanced susceptibility to TB disease in our patient sample. First, there was a significant increase in the frequency of rare NSPs in TB patients compared to the matched controls. Second, in the case of *TLR1*, the TDT analysis revealed that the allele encoding the TLR-248S variant was significantly overrepresented among diseased children (*P* = 0.021). Third, cell biology studies found that several of the *TLR6* variants were deficient in ability to mediate signal transduction. A fourth line of evidence stems from the population genetics analysis. By aligning human *TLR6* coding sequences with the *Bos taurus* homolog (NM_001001159.1), we find that there were 167 nonsynonymous differences and 193 synonymous differences. These numbers vary somewhat depending on the human allele one uses, but the conclusion is robust to this choice–the patient sample has an excess of nonsynonymous variants (*n* = 24) relative to the number of synonymous variants (*n* = 9) compared to the degree of divergence with cow (McDonald–Kreitman test, *X^2^* = 8.39, *P*<0.004). The pattern of polymorphism in *TLR6* in TB patients deviated significantly from neutrality, and the major cause was an excess of rare mutations that would alter the primary amino acid sequence of TLR6. Inasmuch as singleton mutations occurred in significant excess in TB patients, the increase in susceptibility to TB disease associated with these polymorphisms must be substantial. In this regard, we note that before the introduction of widespread anti-MTB drug therapy in the 1950's, the death rate from TB was very high. Hence, the data are broadly consistent with a process whereby variants that reduce the ability to fight infections enter the population by mutation, but have been maintained at a low frequency by the action of natural selection. This process leads to an excess of rare nonsynonymous polymorphism in the human population compared to the number of nonsynonymous fixations between humans and chimpanzees.

Haplotype analysis can be more powerful than SNP analysis for providing insight into the genetic basis of complex traits [42], and provides a fifth line of evidence for the role of TLR polymorphism in TB susceptibility. We found that both rare SNPs and rare haplotypes of *TLR6* also were significantly overrepresented among the TB patients relative to the matched control population. The rare haplotypes present in TB patients do not cluster closely together in the phylogenetic network of TLR6 (Table S3 and Figure S1). Simulations of the neutral coalescent [43], with each sample of size 1,312 having 31 segregating sites (like *TLR6*) were performed to ask how often a random partition into 894 cases and 418 controls would produce a more extreme association with rare alleles in the case group by chance. This test found that only 3 out of 1000 samples had more than the observed number of singletons present in the group of cases than was observed in *TLR6*. The data appear to be consistent with the rare haplotypes being derived independently (and by their rarity, we infer recently) from multiple common haplotypes by single genetic events such as nucleotide substitution or deletion of a short segment of DNA. Insofar as *TLR6* is concerned, this means that TB disease is not associated with a single unique mutation, rather, many independent mutations are associated with enhanced disease susceptibility.

Our findings may bear on a report in the literature suggesting that African Americans may have an enhanced susceptibility to TB disease [44]. We found significant association between common and rare NSPs and increased susceptibility to TB disease in African Americans. The TDT analysis also revealed an association between TLR1 and TB susceptibility. However, these findings were not uniformly the case in the European American and Hispanic populations. It is reasonable to consider what factors may account for the differences between populations. On average, these three populations have quite different recent ancestral histories, which means that they have undoubtedly encountered distinct selective forces, including but not limited to lethal infectious agents such as *M. tuberculosis*. In this regard, we note that Ferwerda et al. [45] recently have hypothesized that certain nonsynonymous amino acid changes polymorphisms in TLR4 rose to high frequency in sub-Saharan Africa because they protect against malaria.

Simply because of their rarity, individual rare variants are not shared across populations, adding to the apparent heterogeneity across population samples in TLR associations. It is very possible that some rare variants increase and some decrease susceptibility to TB. Given that the long-term effective population size of African Americans is larger than the other two populations we studied, one would expect more rare variants to occur in that population. Although several environmental factors are likely operative, and many genes contribute to TB susceptibility, a larger pool of rare variants (in either TLRs or other proteins) could be a factor contributing to an enhanced susceptibility to TB. We note that different selection pressures may also account for the reciprocal allele frequencies at the common variant sites (for example, TLR1-248 and TLR1-602) observed in the African American and European American populations. We also note that several of the amino acid polymorphisms we identified in TLR1 and TLR2 map to regions of these proteins that interact with each other and with lipopeptide, as recently revealed by crystal structure analysis [46].

### Detrimentally Altered Signal-Transduction by Rare Variants of TLR6 and TB Disease Susceptibility

Whether an individual develops TB disease or remains asymptomatic after exposure to the bacterium depends on the ability of the host to effectively kill MTB or sufficiently control its replication. Members of the TLR family of proteins recognize ligands derived from a variety of pathogens and trigger crucial steps in the protective immune response against infectious agents. TLR6 forms a heterodimeric complex with TLR2 to activate macrophages [40], a critical step in defense against MTB. Bulut *et al*. [47] reported that the dominant-negative mutant of mouse TLR6 (Pro691His) inhibited the ability of transfected cells to respond to soluble tuberculosis factor. Hence, it is reasonable to conclude that some of the rare TLR6 variants we identified in the TB patients have altered function. Indeed, the results of our cell biology studies, which focused on the formation of a functional TLR2/TLR6 signaling complex, confirmed this idea. Under a current model of TLR function [15], the proinflammatory signal-transduction cascade that protects the host from disease caused by MTB may be detrimentally altered in individuals with rare TLR6 variants, thereby leading to enhanced likelihood of clinical disease. Studies are underway to examine this issue in the context of primary cells obtained from patients and controls with known *TLR6* genotypes.

### Rare Variants of Proteins Involved in Human Innate Immune Function and Infectious Disease Susceptibility

It is generally accepted that human susceptibility to TB disease is a complex genetic trait [4], [5]. Substantial controversy exists in human medical genetics regarding the contribution of common and rare mutations to susceptibility to complex diseases [48], [49]. One school of thought favors the idea that common genetic diseases are associated with polymorphisms that are present in relatively high frequency in a population (common disease/common variant model). Another school of thought argues that rare mutations play a much more important role in susceptibility to common diseases than usually assumed, and interest in this area is accelerating [50]–[52]. The results of our study support both arguments. While we do find common variants that display significant association with TB susceptibility, we also present evidence that rare variants of proteins that detrimentally alter innate host defense are important contributors to the outcome of the interaction between a bacterial pathogen and the infected individual. This theme is echoed by the finding that a large array of distinct rare variants of the IL-12 receptor β1 result in enhanced susceptibility to infections caused by bacteria such as *Salmonella* spp., *M. tuberculosis* BCG, and environmental mycobacteria [53]–[55]. Moreover, we note that well over 200 otherwise rare mutations can cause X-linked chronic granulomatous disease [56], an abnormality characterized by altered ability of polymorphonuclear leukocytes to kill several bacterial and fungal pathogens. Similarly, Picard *et al*. [57] recently reported the occurrence of rare mutations in IRAK-4 that confer susceptibility to pyogenic bacterial infections.

Thus far, human TB susceptibility studies have been conducted largely by association studies that examine the effect of common mutations in patients and controls [4], [5]. One important practical consequence of the involvement of many distinct rare variants in host defense is that it likely will be necessary to completely sequence candidate susceptibility genes in large samples of affected individuals and control subjects to identify disease association.

In summary, by discovering a link between TLR variants, altered TLR signal transduction, and enhanced susceptibility to TB disease, our findings provide strong impetus to analyze the relationship between nucleotide sequence variation in other human candidate genes, and susceptibility to infectious agents. Our study was designed to investigate the association of TLR variants with TB disease in only one geographic area that was purposely defined narrowly. Our findings may not be generally applicable to all TB patients. Indeed, there is substantial variance in the results of TB susceptibility studies reported in the literature for mice and humans [4], [5], [58]. In the case of humans, this variance may be caused by differences in ethnicity, environmental factors such as nutrition, and exposure to nontuberculous mycobacteria. Another variable that may, in principle, influence host-pathogen interaction is the relative frequency of distinct clones of MTB circulating in the human population under study. Although strains of MTB are, on average, relatively closely related in overall chromosomal character, many strain-to-strain genetic differences have been described, and the frequency of occurrence of distinct clones differs geographically [59]–[62]. In addition, evidence is accumulating that strains can differ significantly in virulence [63]. The possibility that different host genotypes have differential susceptibility across different MTB strains remains to be tested. It is a formal possibility that the mechanisms used by MTB strains to overcome host immunity differ in a strain-dependent fashion, or differ depending on human genotype/strain genotype combination. Clearly, studies analogous to the one we report here need to be conducted in human populations that differ in ethnicity and age from the one we investigated. It is also reasonable to speculate that rare TLR variants influence human susceptibility to disease caused by other infectious agents.

## Materials and Methods

### Population-Based Adult Case-Control Study

This study was based on analysis of 1,312 HIV-seronegative adults living in Houston, Texas, enrolled in a population-based study of TB [7], [64]. Patients (*n* = 894) with TB disease included 339 African Americans (mean age±SD, 46.5±12.8; male, 68.7%; extrapulmonary TB, 15.6%), 180 European Americans (mean±SD, 51.7±13.6; male, 75.0%; extrapulmonary TB, 11.1%), and 375 Hispanics (mean±SD, 42.3±17.2; male, 63.5%; extrapulmonary TB, 14.7%). All patients were identified by either MTB culture positivity (92.8%) or clinical improvement (7.2%) in response to antimycobacterial treatment without a history of immunosuppressive condition. Extra-pulmonary TB was defined as involvement of an extrathoracic site, or the thoracic pleura or lymph nodes according to the official statement of the American Thoracic Society and the Centers for the Disease Control and Prevention (CDC). Patients with both pulmonary and extra-pulmonary TB were classified as extra-pulmonary. None of the patients were known to be genetically related.

The control sample (*n* = 418) consisted of 194 African Americans (mean±SD, 45.5±10.6; male, 75.8%), 110 European Americans (mean age±SD, 50.0±10.0; male, 67.3%) and 114 Hispanics (mean age±SD, 43.4±11.0; male, 58.8%) without a history of TB disease, autoimmune diseases, and other infectious diseases. These individuals either lived in the same neighborhood as a TB patient or were health care workers. All control subjects were followed for at least 6 months to confirm their clinical status. In this study, ethnicity was determined by self-identification. European Americans were defined as non-Hispanic, white individuals. Written informed consent was obtained from all study participants for the studies performed herein. The study was approved by the Institutional Review Board at Baylor College of Medicine.

Cut-off lines used for rare and common polymorphisms are usually 1% to 5% depending on the study. We used 3% as a cut-off value because the sample sizes in our adult case-control study ranged from 110 to 375 in different ethnic groups of cases and controls. If a SNP had an allele frequency of 3% in one ethnic group, it will have at least 6.6 alleles in the smallest group having 110 individuals (e.g. European American controls). This is statistically feasible for a 2×2 Chi-square test.

### Transmission/Disequilibrium Test (TDT) within the Families of Children with TB

To avoid potential bias in the adult case-control study caused by population substructures or control selection, a separate family-based association study using TDT was conducted to provide additional support for the detected TLR1 variant associations. Ninety-nine nuclear families consisting of one child with TB and two biological parents from the greater Houston area were studied.

### Sequencing of *TLR* Genes

Genomic DNA was prepared from peripheral blood leukocytes by standard phenol-chloroform extraction methods. Full-coding region sequencing was conducted on all five *TLR* genes (*TLR1*, *TLR2*, *TLR4*, *TLR6*, and *TLR10*) in the 1,312 subjects. PCR products used for sequencing were obtained by amplifying genomic DNA with the primers shown in Table S4. DNA sequencing was conducted with an ABI 3730 instrument. The software used for initial data analysis was Sequencher (Gene Codes, Ann Arbor, MI). All raw data were assembled and analyzed by two individuals independently and blinded to the analyses performed by the other. The data were compared, and the few discrepancies were resolved. This procedure, although tedious, resulted in a data set of unusually high quality.

### Statistical Analysis of the Adult Subject Data

Within each ethnic grouping, the 179 SNP genotypes were examined for hidden population subdivision using the program STRUCTURE 2.2 [65]. Seeing no evidence for latent structure, Hardy Weinberg tests were performed for each SNP that presented 3 genotypic classes. The homogeneity of frequencies of genotypes between the cases and controls in each ethnic group were tested by using a Chi-square test for two-by-two and two-by-three contingency tables with SAS (version 8.0, SAS Institute; Cary, NC). *P*-values were corrected for multiple comparisons by the formula (*P_corr_* = 1– [1–*P*]^n^, with *P* being the uncorrected *P* value, and n the number of comparisons). A *P_corr_* less than 0.05 was considered to be statistically significant. The significant tests reported here were also significant by standard Bonferroni correction.

### Plasmid Constructs and Site-Directed Mutagenesis

The reference allele and the most common allele of human *TLR6*, haplotype h2 (Figure S1 and Table S3), were amplified from genomic DNA and cloned into pCR-XL-TOPO (Invitrogen, Carlsbad, CA). Rare allelic variants of *TLR6* and the allele of *TLR6* encoding a dominant-negative form (Pro680His) of the receptor were made using the QuickChange Multi Site-Directed Mutagenesis system (Stratagene, La Jolla, CA). The analogous dominant negative mutation in mouse TLR6 (Pro691His) was reported and characterized previously [41]. *Spe*I restriction fragments made from the pCR-TOPO-XL-TLR6 clones containing the *TLR6* allelic variants were subcloned into the pUNO-hTLR6 cDNA expression vector (InvivoGen, San Diego, CA). The *TLR6* allelic variant, h19 (Figure S1), characterized by a 5 bp deletion at codon 460 and 461, had additional common polymorphisms of which one occurred outside of the *Spe*I sites. Thus, the *TLR6* gene for the 5 bp deletion variant was amplified from genomic DNA and cloned into the *Age*I and *Nhe*I sites of pUNO-hTLR6. All constructs were verified by sequencing of the complete *TLR6* with an ABI PRISM 3700 DNA Analyzer (Applied Biosystems, Foster City, CA). The β-actin-*Renilla* luciferase construct used as a transfection control was generously provided by C.B. Wilson and A.M. Hajjar, University of Washington. The NF-κB reporter plasmid, ELAM-1-firefly luciferase, and the human TLR2 cDNA expression construct, gD.TLR2, have been described previously [31], [33].

### Cell Culture, Transfections, and Dual Luciferase Reporter Assays

The human embryonic kidney (HEK) 293 cell line (CRL-1573) was purchased from the American Type Culture Collection (Manassas, VA) and grown at 37°C in 5% CO_2_ in DMEM medium supplemented with 10 mM HEPES, 100 IU penicillin, 100 μg streptomycin/ml, and 10% fetal bovine serum (Invitrogen, Carlsbad, CA). HEK 293 cells were seeded into 24-well plates (1×10^5^ cells/well) the day before transfection. Cells were transiently transfected with 0.3 μg of the specified *TLR6* expression construct and 0.006 μg of plasmid gD.TLR2, 0.091 μg of NF-κB reporter, and 0.003 μg of β-actin-*Renilla* luciferase plasmid. Transfections were performed using FuGene 6 transfection reagent (Roche, Indianapolis, IN) as recommended by the manufacturer. Twenty-four hours post transfection, cell lysates were prepared and luciferase activity for each reporter construct was determined using the Dual-Luciferase Reporter assay system (Promega, Madison, WI) following the manufacturer's specifications.

## Supporting Information

Figure S1 Haplotype analysis of TLR6 variants. Thirty-two haplotypes were identified or inferred based on the segregation sites in the coding region of TLR6. The reference sequence from the database (GenBank accession no. AB020807) was designated as h0. Six of 32 haplotypes were characterized by the presence of only 1 mutation, whereas the other 27 haplotypes were differentiated by at lease 2 mutations (Table S3). The haplotypes tend to cluster in two major clades, h0 and h2. The chimpanzee lineage was located between the two major clades, suggesting that h0 and h2 are probably quite ancient. Three cycles are present in the haplotype network (h2-h7-h10-h2; h2-h1-h6-h8-h2; h2-h22-chimp-h2), suggesting that intragenic recombination or repeated mutation occurred in the evolutionary history of TLR6.
(1.17 MB TIF)

Table S1 Genetic variants in human TLR1, 2, 4, 6, 10 genes
(0.10 MB PDF)

Table S2 Associations between common nonsynonymous variants of TLR1, 4, 6, 10 genes with TB and Extrapulmonary TB (EPTB) disease
(0.21 MB DOC)

Table S3 Haplotype analysis of TLR6 genetic variants
(0.04 MB PDF)

Table S4 Oligonucleotide primers used to amplify and sequence TLR1, 2, 4, 6, and 10
(0.06 MB PDF)
