# Supplementary figures and images for: Full-Exon Resequencing Reveals Toll-Like Receptor Variants Contribute to Human Susceptibility to Tuberculosis Disease
Source: PLoS One. 2007 Dec 19;2(12):e1318. doi: 10.1371/journal.pone.0001318 (PMC2117342; doi:10.1371/journal.pone.0001318)

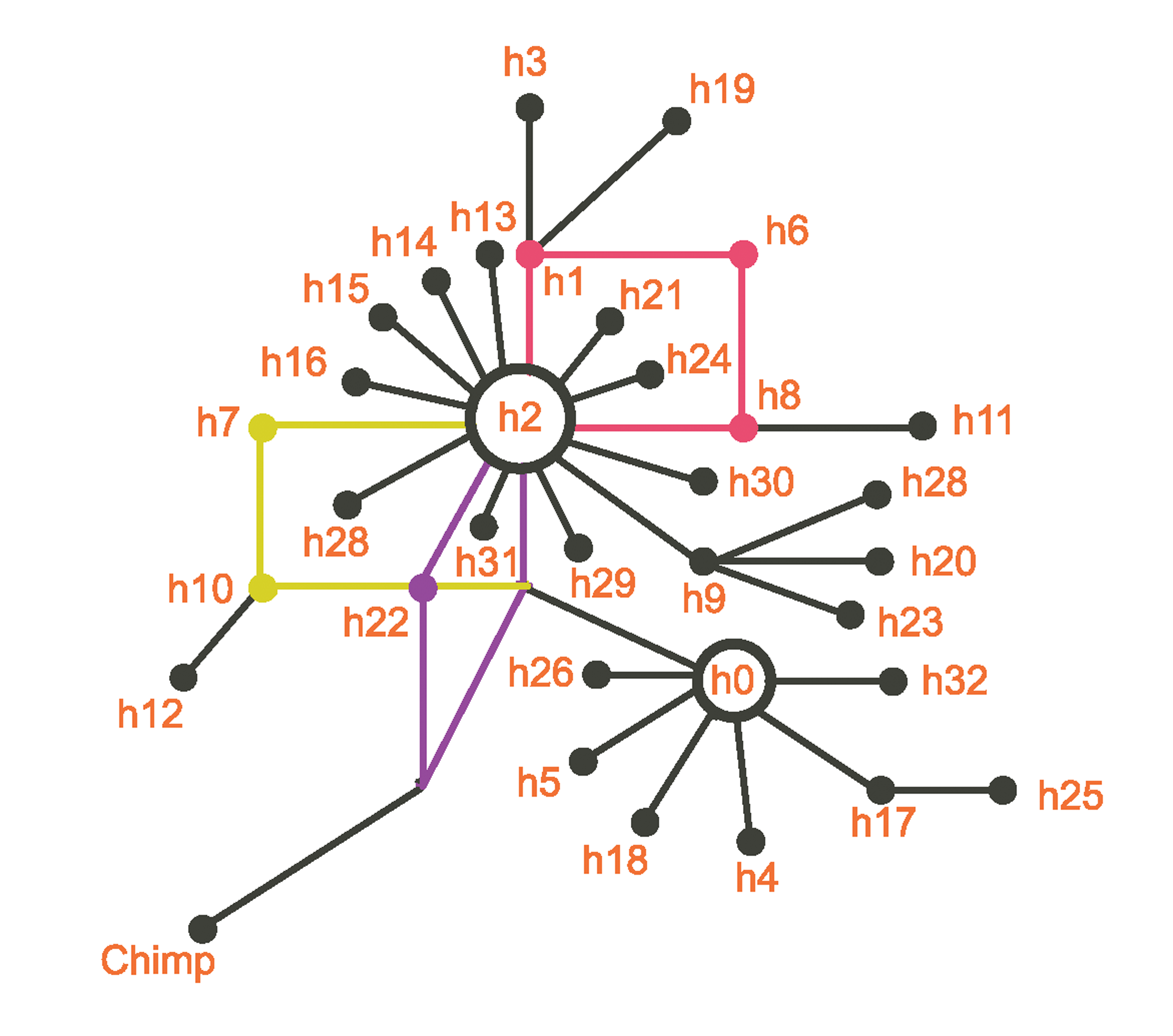

Supplement: Figure S1 — Haplotype analysis of TLR6 variants. Thirty-two haplotypes were identified or inferred based on the segregation sites in the coding region of TLR6. The reference sequence from the database (GenBank accession no. AB020807) was designated as h0. Six of 32 haplotypes were characterized by the presence of only 1 mutation, whereas the other 27 haplotypes were differentiated by at lease 2 mutations (Table S3). The haplotypes tend to cluster in two major clades, h0 and h2. The chimpanzee lineage was located between the two major clades, suggesting that h0 and h2 are probably quite ancient. Three cycles are present in the haplotype network (h2-h7-h10-h2; h2-h1-h6-h8-h2; h2-h22-chimp-h2), suggesting that intragenic recombination or repeated mutation occurred in the evolutionary history of TLR6. (1.17 MB TIF) [file pone.0001318.s001.tif]
