# Supplementary material for: Full-Exon Resequencing Reveals Toll-Like Receptor Variants Contribute to Human Susceptibility to Tuberculosis Disease
Source: PLoS One. 2007 Dec 19;2(12):e1318. doi: 10.1371/journal.pone.0001318 (PMC2117342; doi:10.1371/journal.pone.0001318)
Supplement: Table S1 — Genetic variants in human TLR1, 2, 4, 6, 10 genes (0.10 MB PDF) [file pone.0001318.s002.pdf]

Table S1. Genetic variants in human *TLR1, 2, 4, 6, 10* genes

SNPs	Genotype	African American Cases *n* = 339	African American Controls *n* = 194	European American Cases *n* = 180	European American Controls *n* = 110	Hispanic cases *n* = 375	Hispanic controls *n* = 114
**TLR1**							
36 CTC→TTC	CC			180	109		
Leu→Phe	CT			0	1		
38 CAC→CAT	CC	339	192			370	114
His→His	CT	0	2			5	0
57 ATA→ATG	AA			180	109	373	113
Ile→Met	AG			0	1	2	1
80 AGA→ACA	GG	334	194	161	101	366	109
Arg→Thr	GC	5	0	14	9	9	5
	CC	0	0	5	0	0	0
118 CAC→TAC	CC	286	179	180	109	371	112
His→Tyr	CT	53	15	0	1	4	2
137 AAT→ACT	AA	338	194				
Asn→Thr	AC	1	0				
138 ATG→CTG	AA	338	194				
Met→Leu	AC	1	0				
193 CAC→CAT	CC					374	114
His→His	CT					1	0
243 AAT→ATT	AA	338	194				
Asn→Ile	AT	1	0				
248 AAT→AGT	GG	240	116	1	3	100	24
Asn→Ser	AG	68	61	28	31	187	52
	AA	31	17	151	76	88	38
250 ACC→AC-	CC			179	110		
Thr→FS	Del C			1	0		
305 CAC→CTC	AA	309	175	174	104	354	110
His→Leu	AT	30	17	6	6	21	4
	TT	0	2	0	0	0	0
315 CCG→CTG	CC	337	194				
Pro→Leu	CT	2	0				
335 GGT→GCT	GG	337	194				
Gly→Ala	GC	2	0				
352 CAT→AAT	CC	308	189				
His→Asn	CA	31	5				

SNPs	Genotype	African American Cases *n* = 339	African American Controls *n* = 194	European American Cases *n* = 180	European American Controls *n* = 110	Hispanic cases *n* = 375	Hispanic controls *n* = 114
435 CTT→CT-	TT					374	114
Leu→FS	Del T					1	0
506 TCG→TCA	AA	206	86	8	8	112	24
Ser→Ser	AG	110	82	52	31	179	53
	GG	23	26	120	71	84	37
528 TGT→TGC	TT	299	172	179	110	371	114
Cys→Cys	TC	38	22	1	0	4	0
	CC	2	0	0	0	0	0
541 CAA→CCA	AA	339	193	179	110		
Gln→Pro	AC	0	1	1	0		
542 GTA→GCA	TT	332	193			374	114
Val→Ala	TC	7	1			1	0
548 GAG→AAG	GG					374	114
Glu→Lys	GA					1	0
587 GTT→GGT	TT	330	191	179	110	374	113
Val→Gly	TG	9	3	1	0	1	1
602 AGC→ATC	TT	272	120	12	14	272	61
Ser→Ile	TG	63	61	61	33	83	39
	GG	4	13	107	63	20	14
619 ACC→ACT	CC	338	194				
Thr→Thr	CT	1	0				
631 CTC →CGC	TT					374	114
Leu→Arg	TG					1	0
651 GTG→GCG	TT	337	194				
Val→Ala	TC	2	0				
656 TTG→TTA	GG	322	190				
Leu→Leu	GA	16	4				
	AA	1	0				
676 GGC→GGT	CC	337	193				
Gly→Gly	CT	2	1				
693 TCC→GCC	TT	338	194				
Ser→Ala	TG	1	0				
720 CAT→CCT	AA	338	193				
His→Pro	AC	1	1				
733 CCC→CTC	CC	332	193				
Pro→Leu	CT	7	1				

SNPs	Genotype	African American Cases *n* = 339	African American Controls *n* = 194	European American Cases *n* = 180	European American Controls *n* = 110	Hispanic cases *n* = 375	Hispanic controls *n* = 114
**TLR2**							
26 GCT→GTT	CC	338	194				
Ala→Val	CT	1	0				
38 GGC→GGA	CC	335	193			373	114
Gly→Gly	CA	4	1			2	0
80 GCT→GCCT	TT	339	193				
INS C	INS C	0	1				
82 GTG→GTA	GG	338	194				
Val→Val	GA	1	0				
83 CTG→TTG	CC	338	194				
Leu→Leu	CT	1	0				
91 ATA→GTA	AA	336	194				
Ile→Val	AG	3	0				
119 TGG→TGA	GG	338	194				
Trp→Ter	GA	1	0				
120 TTC→GTC	TT	338	194				
Phe→Val	TG	1	0				
199 AAT→AAC	TT	46	29	55	41	133	18
Asn→Asn	TC	165	100	90	47	191	80
	CC	128	65	35	22	51	16
217 TTT→TCT	TT			179	110	374	114
Phe→Ser	TC			1	0	1	0
231 GAT→GGT	AA	338	194				
Asp→Gly	AG	1	0				
269 GTT→GTA	TT			179	110		
Val→Val	TA			1	0		
411 ACT→ATT	CC					367	114
Thr→Ile	CT					8	0
411 ACT→ACC	TT					373	114
Thr→Thr	TC					2	0
415 TTG→TGG	TT			179	110		
Leu→Trp	AG			1	0		
427 TCT→TTT	CC	339	192			373	105
Ser→Phe	CT	0	2			2	9
447 CGA→CAA	GG	337	194			373	113
Arg→Gln	GA	2	0			2	1

SNPs	Genotype	African American Cases *n* = 339	African American Controls *n* = 194	European American Cases *n* = 180	European American Controls *n* = 110	Hispanic cases *n* = 375	Hispanic controls *n* = 114
447 CGA→TGA	CC	338	194	179	110		
Arg→Ter	CT	1	0	1	0		
450 AGT→AGC	TT	299	169	151	101	312	100
Ser→Ser	TC	38	25	24	9	62	14
	CC	2	0	5	0	1	0
455 ATT→GTT	AA			179	110		
Ile→Val	AG			1	0		
456 CCC→TCC	CC	338	194				
Pro→Ser	CT	1	0				
486 AGA→ACA	GG	337	194				
Arg→Thr	GC	2	0				
526 AAG→AAC	GG	338	194				
Lys→Asn	GC	1	0				
541 TTC→TTT	CC	333	185	165	104	365	111
Phe→Phe	CT	6	8	15	6	10	3
	TT	0	1	0	0	0	0
542 CTC→CTG	CC	332	183				
Leu→Leu	CG	7	11				
556 ATT→ACT	TT			180	109		
Ile→Thr	TC			0	1		
571 CGT→CAT	TT	337	190			374	113
Arg→His	TC	2	4			1	1
579 CGC→CAC	GG	334	192				
Arg→His	GA	5	2				
631 CCC→CAC	CC	338	194				
Pro→His	CA	1	0				
650 CGG→CAG	GG			180	109		
Arg→Gln	GA			0	1		
707 TTT→TTC	TT	338	192	178	110	355	96
Phe→Phe	TC	1	2	2	0	20	18
738 GAG→CAG	GG	336	192				
Glu→Gln	GC	3	2				
753 CGG→CAG	GG	337	194	171	105	374	110
Arg→Gln	GA	2	0	9	5	1	4
762 CTG→CTA	GG	338	192				
Leu→Leu	GA	1	2				

SNPs	Genotype	African American Cases n = 339	African American Controls n = 194	European American Cases n = 180	European American Controls n = 110	Hispanic cases n = 375	Hispanic controls n = 114
781 GCG→GCA	GG	336	192	185	109	298	98
Ala→Ala	GA	3	2	0	1	75	16
	AA	0	0	0	0	2	0
**TLR4**							
39 CAA→CAC	AA	338	194				
Gln→His	AC	1	0				
46 TAC→TGC	AA	337	193				
Tyr→Cys	AG	2	1				
61 CTG→CCG	TT			180	109		
Leu→Pro	TC			0	1		
105 TCT→TCC	TT	249	150			366	114
Ser→Ser	TC	86	43			9	0
	CC	4	1			0	0
113 CCC→CCT	CC	335	193				
Pro→Pro	CT	4	1				
115 CAG→CGG	AA	339	193				
Gln→Arg	AG	0	1				
145 CCC→CCA	CC	338	194	180	109		
Pro→Pro	CA	1	0	0	1		
146 ATT→ACT	TT	339	193				
Ile→Thr	TC	0	1				
163 CAA→CAG	AA	332	188			375	112
Gln→Gln	AG	6	6			0	2
	GG	1	0			0	0
175 ACC→GCC	AA			180	109		
Thr→Ala	AG			0	1		
188 CAA→CGA	AA	337	194				
Gln→Arg	AG	2	0				
222 GCA→ACA	GG	338	194				
Ala →Thr	GA	1	0				
244 AAA→AGA	AA			179	110		
Lys→Arg	AG			1	0		
281 TGC→TAC	GG			180	108	374	114
Cys→Tyr	GA			0	2	1	0
299 GAT→GGT	AA	281	157	159	95	353	106
Asp→Gly	AG	57	36	20	14	22	8
	GG	1	1	1	1	0	0

SNPs	Genotype	African American Cases *n* = 339	African American Controls *n* = 194	European American Cases *n* = 180	European American Controls *n* = 110	Hispanic cases *n* = 375	Hispanic controls *n* = 114
301 ATT→ACT	TT	338	193				
Ile→Thr	TC	1	1				
354 AAA→AAG	AA	339	192	178	107	373	114
Lys→Lys	AG	0	2	2	3	2	0
361 AAC→GAC	AA	338	194				
Asn→Asp	AG	1	0				
385 TTG→TTT	GG	337	194				
Leu→Phe	GT	2	0				
399 ACC→ATC	CC	325	178	161	97	357	108
Thr→Ile	CT	14	16	18	12	18	6
	TT	0	0	1	1	0	0
443 TTC→TTT	CC	337	194	179	110	374	114
Phe→Phe	CT	2	0	1	0	1	0
474 GAA→AAA	GG	308	184	180	109	372	114
Glu→Lys	GA	30	10	0	1	3	0
	AA	1	0	0	0	0	0
510 CAG→CAT	GG	326	183	179	110	374	113
Gln→His	GT	13	11	1	0	1	1
562 CAG→CGG	AA					374	114
Gln→Arg	AG					1	0
593 TGG→TGA	GG					375	111
Trp→Ter	GA					0	3
634 ATT→ACT	TT	337	194			374	114
Ile→Thr	TC	2	0			1	0
640 AGT→AGA	TT					374	114
Ser→Arg	TA					1	0
653 AAG→AAA	GG	337	188	177	108	374	114
Lys→Lys	GA	2	6	3	2	1	0
670 GGT→GAT	GG	338	194				
Gly→Asp	GA	1	0				
682 AGC→AGA	CC	338	194				
Ser→Arg	CA	1	0				
731 CGA→TGA	CC	339	193				
Arg→Ter	CT	0	1				
763 CGT→CAT	GG					373	114
Arg→His	GA					2	0
804 CGG→TGG	CC	338	193			374	114
Arg→Trp	CT	1	1			1	0

SNPs	Genotype	African American Cases *n* = 339	African American Controls *n* = 194	European American Cases *n* = 180	European American Controls *n* = 110	Hispanic cases *n* = 375	Hispanic controls *n* = 114
804 CGG→CAG	GG					374	114
Arg→Gln	GA					1	0
817 GAT→GAG	TT			179	110		
Asp→Glu	TG			1	0		
834 CAG→AAG	CC					375	111
Gln→Lys	CA					0	3
838 TCT→TTT	CC			179	110		
Ser→Phe	CT			1	0		
**TLR6**							
30 GAC→AAC	GG	337	194				
Asp→Asn	GA	2	0				
120 ATT→ACT	TT	283	176	179	108	372	114
Ile→Thr	TC	52	18	1	2	3	0
	CC	4	0	0	0	0	0
125 CAT→CTT	AA					374	114
His→Leu	AT					1	0
156 ATG→GTG	AA	338	194				
Met→Val	AG	1	0				
194 CTG→CCG	TT	317	174			374	114
Leu→Pro	TC	22	20			1	0
209 TTC→TTT	CC					374	114
Phe→Phe	CT					1	0
227 AAT→AAC	TT	337	194				
Asn→Asn	TC	2	0				
247 AGA→AAA	GG	328	191			373	114
Arg→Lys	GA	11	3			2	0
249 TCA→CCA	TT	3	7	31	26	10	5
Ser→Pro	TC	47	50	88	46	74	31
	CC	289	137	61	38	291	78
251 TTA→TAA	TT	378	194				
Leu→Ter	TA	1	0				
255 ACC→ATC	CC	337	194			374	114
Thr→Ile	CT	2	0			1	0
257 AAC→GAC	AA					374	114
Asn→Asp	AG					1	0
287 ACA→ACG	AA	310	172			373	114
Thr→Thr	AG	29	22			2	0
298 TTA→CTA	TT	338	194				
Leu→Leu	TC	1	0				

SNPs	Genotype	African American Cases *n* = 339	African American Controls *n* = 194	European American Cases *n* = 180	European American Controls *n* = 110	Hispanic cases *n* = 375	Hispanic controls *n* = 114
327 GTG→ATG	GG	323	187			373	114
Val→Met	GA	16	7			2	0
345 CAC→TAC	CC					374	114
His→Tyr	CT					1	0
357 TTG→CTG	TT					373	114
Leu→Leu	TC					2	0
361 ACC→ACG	CC	50	54	77	45	168	30
Thr→Thr	CG	147	71	81	50	169	73
	GG	142	69	22	15	38	11
388 GGA→GAA	GG	339	193				
Gly→Glu	GA	0	1				
399 ACG→ATG	CC	339	193				
Thr→Met	CT	0	1				
421 AAA→AAG	AA	40	49	76	44	170	27
Lys→Lys	AG	147	70	81	51	169	76
	GG	152	75	23	15	36	11
427 GTT→GCT	TT	333	191	175	107	317	111
Val→Ala	TC	6	3	5	3	53	3
	CC	0	0	0	0	5	0
458 CAC→CGC	AA	337	194	180	109		
His→Arg	AG	2	0	0	1		
460/461 5bp Del	AATAA	338	194				
Del AATAA-Fs	DEL	1	0				
465 GTT→ATT	GG	278	179	179	110	373	113
Val→Ile	GA	59	15	1	0	2	1
	AA	2	0	0	0	0	0
474 GCT→ACT	GG	290	180	178	108	371	108
Ala→Thr	GA	46	13	2	2	4	6
	AA	3	1	0	0	0	0
567 TAT→TGT	AA			184	110		
Tyr→Cys	AG			1	0		
572 CTA→CTG	TT	338	194				
Leu→Leu	TG	1	0				
592 GGT→GTT	GG	337	194				
Gly→Val	GT	2	0				
644 GCT→GCG	TT	297	167	86	58	341	88
Ala→Ala	TG	39	21	84	41	31	24
	GG	3	6	10	11	3	2

SNPs	Genotype	African American Cases *n* = 339	African American Controls *n* = 194	European American Cases *n* = 180	European American Controls *n* = 110	Hispanic cases *n* = 375	Hispanic controls *n* = 114
690 AAC→ACC	AA	336	194			374	114
Asn→Thr	AC	3	0			1	0
756 ACG→ATG	CC			178	110		
Thr→Met	CT			2	0		
777 ATT→GTT	AA					375	113
Ile→Val	AG					0	1
**TLR10**							
13 ATT→ATA	TT	116	101	119	80	212	80
Ile→Ile	TA	154	67	57	30	140	31
	AA	69	26	4	0	23	3
51 ACG→ACA	GG	211	152	161	104	262	96
Thr→Thr	GA	116	32	18	6	103	17
	AA	12	10	1	0	10	1
59 CTC→ATC	CC					374	114
Leu→Ile	CA					1	0
74 CTG→TTG	CC			180	109		
Leu→Leu	CT			0	1		
90 AAA→AAG	AA	276	169	179	109	368	113
Lys→Lys	AG	63	25	1	1	7	1
96 AAG→AGG	AA	338	194	180	109	374	114
Lys→Arg	AG	1	0	0	1	1	0
127 AAT→AGT	AA	337	194				
Asn→Ser	AG	2	0				
132 ATG→ACG	TT			180	102	374	114
Met→Thr	TC			0	8	1	0
163 GCT→TCT	GG	313	182	163	106	268	97
Ala→Ser	GT	26	12	16	4	100	17
	TT	0	0	1	0	7	0
213/214 DEL TT	WT					374	114
Leu→FS	DEL					1	0
241 AAT→CAT	AA	79	47	78	52	150	61
Asn→His	AC	172	100	85	52	182	41
	CC	88	47	17	6	43	12
257 GAC→GAT	CC	338	193				
Asp→Asp	CT	1	1				
258 GAC→GAA	CC	336	191				
Asp→Glu	CG	3	3				
261 CTT→GTT	TT	336	193			374	114
Leu→Val	TC	3	1			1	0

SNPs	Genotype	African American Cases n = 339	African American Controls n = 194	European American Cases n = 180	European American Controls n = 110	Hispanic cases n = 375	Hispanic controls n = 114
298 GTA→ATA	GG	317	183	163	108	281	97
Val→Ile	GA	22	11	17	2	94	17
303 AAA→AAG	AA	181	112	133	89	322	98
Lys→Lys	AG	130	66	45	21	53	14
	GG	28	16	2	0	0	2
326 ATG→ACG	TT	325	186	163	106	278	96
Met→Thr	TC	14	8	16	4	96	18
	CC	0	0	1	0	1	0
344 CCG→CCT	GG	189	112	117	80	189	79
Pro→Pro	GT	134	69	57	26	159	26
	TT	16	13	6	4	27	9
361 ACA→ACG	AA	330	191				
Thr→Thr	AG	9	3				
369 ATC→CTC	AA	72	43	76	58	148	62
Ile→Leu	AC	179	103	89	46	185	42
	CC	88	48	15	6	42	10
370 CAA→TAA	CC	323	188			373	113
Gln→Ter	CT	16	6			2	1
380 AAT→AAC	TT	336	193				
Asn→Asn	TC	3	1				
381 GGC→GAC	GG	315	190	179	108	366	111
Gly→Asp	GA	24	4	1	2	9	3
428 CTG→CCG	TT	333	191				
Leu→Pro	CC	6	3				
431 AAT→AAC	TT			180	109		
Asn→Asn	CC			0	1		
433 TTG→CTG	TT					374	114
Leu→Leu	TC					1	0
471 CTA→TTA	CC			179	110		
Leu→Leu	CT			1	0		
473 ATT→ACT	TT	338	193	176	108	364	113
Ile→Thr	TC	1	1	4	2	11	1
519 GCG→GTG	CC	337	194				
Ala→Val	CT	2	0				
520 GGA→AGA	GG	338	194				
Gly→Arg	GA	1	0				
525 CGG→TGG	CC	323	190	175	107	366	112
Arg→Trp	CT	16	4	5	3	9	2

SNPs	Genotype	African American Cases n = 339	African American Controls n = 194	European American Cases n = 180	European American Controls n = 110	Hispanic cases n = 375	Hispanic controls n = 114
544 ATG→ACG	TT					373	114
Met→Thr	TC					2	0
564 TTA→GTA	TT					374	114
Leu→Val	TG					1	0
570 CAC→CAT	CC					370	114
His→His	CT					5	0
603 CTG→TTG	TT	287	169	178	110	369	112
Leu→Leu	TG	52	25	2	0	6	2
612 CAA→CAG	AA	338	194				
Gln→Gln	AG	1	0				
724 CAT→CAC	TT	197	119	119	80	195	86
His→His	TC	127	63	55	25	154	22
	CC	15	12	6	5	26	6
736 TAT→TGT	AA					372	114
Tyr→Cys	AG					3	0
742 TAT→TAC	TT	336	191			375	113
Tyr→Tyr	TC	3	3			0	1
752 AAA→AGA	AA	338	194			374	114
Lys→Arg	AG	1	0			1	0
775 ATT→GTT	AA	320	167	138	85	298	93
Ile→Val	AG	18	24	38	22	73	20
	GG	1	3	4	3	4	1
799 CGA→CAA	GG	339	193	180	109	372	112
Arg→Gln	GA	0	1	0	1	3	2
809 GAT→GAC	TT	191	124	121	78	190	79
Asp→Asp	TC	136	60	54	28	158	26
	CC	12	10	5	4	27	9
