# Supplementary material for: Full-Exon Resequencing Reveals Toll-Like Receptor Variants Contribute to Human Susceptibility to Tuberculosis Disease
Source: PLoS One. 2007 Dec 19;2(12):e1318. doi: 10.1371/journal.pone.0001318 (PMC2117342; doi:10.1371/journal.pone.0001318)
Supplement: Table S2 — Associations between common nonsynonymous variants of TLR1, 4, 6, 10 genes with TB and Extrapulmonary TB (EPTB) disease (0.21 MB DOC) [file pone.0001318.s003.doc]

| **Table S2. Associations between common nonsynonymous variants of *TLR1, 4, 6, 10* genes with TB and Extrapulmonary TB (EPTB) disease** |  | **African Americans** | | **Caucasians** | | **Hispanics** | |
| --- | --- | --- | --- | --- | --- | --- | --- |
| **Common**  **variants**^*^ | **Genotypes** | Cases  *n* = 339 | Controls  *n* = 194 | Cases  *n* = 180 | Controls  *n* = 110 | Cases  *n* = 375 | Controls  *n* = 114 |
| ***TLR 1*** |  |  |  |  |  |  |  |
| R80T | GG | 334 | 194 | 161 (89.4) †/ 15^#^ | 101 (91.8) | 366 | 109 |
| AGA→ACA | GC | 5 | 0 | 14 (7.8) / 5 | 9 (8.2) | 9 | 5 |
|  | CC | 0 | 0 | 5 (2.8) / 0 | 0 (0) | 0 | 0 |
| EPTB | CC+GC vs GG |  |  | **OR = 1.32** | *P* = 0.507 |  |  |
|  |  |  |  | **OR = 3.74 *** | *P* = 0.026 |  |  |
| H118Y | CC | 286 (84.4) / 42 | 179 (92.3) | 180 | 109 | 371 | 112 |
| CAC→TAC | CT | 53 (15.6) / 11 | 15 (7.7) | 0 | 1 | 4 | 2 |
| EPTB | CT vs CC | **OR = 2.21** | *P* = 0.009 |  |  |  |  |
|  |  | **OR = 3.13** | *P* = 0.006 |  |  |  |  |
| N248S | GG | 240 (70.8) / 37 | 116 (59.8) | 1 (0.6) / 0 | 3 (2.7) | 100 (26.7) / 21 | 24 (21.1) |
| AAT→AGT | AG | 68 (20.1) / 10 | 61 (31.4) | 28 (15.5) / 2 | 31 (28.2) | 187 (49.9) / 19 | 52 (45.6) |
|  | AA | 31 (9.1) / 6 | 17 (8.8) | 151 (83.9) / 18 | 76 (69.1) | 88 (23.4) / 15 | 38 (33.3) |
| EPTB | GG vs AG+AA | OR = 1.63 | *P* = 0.009 | OR = 0.20 | *P* = 0.123 | **OR = 1.36** | *P* = 0.228 |
|  |  | OR = 1.55 | P = 0.18 |  |  | **OR = 2.32** | *P* = 0.018 |
| H305L | AA | 309 (91.2) / 44 | 175 (90.2) | 174 | 104 | 354 | 110 |
| CAC→CTC | AT | 30 (8.8) / 9 | 17 (8.8) | 6 | 6 | 21 | 4 |
|  | TT | 0 (0) / 0 | 2 (1.0) | 0 | 0 | 0 | 0 |
| EPTB | AT+TT vs AA | OR = 0.89 | *P* = 0.717 |  |  |  |  |
|  |  | OR = 1.88 | *P* = 0.144 |  |  |  |  |
| H352N | CC | 308 (90.9) / 50 | 189 (97.4) |  |  |  |  |
| CAT→AAT | CA | 31 (9.1) / 3 | 5 (2.6) |  |  |  |  |
| EPTB | CA vs CC | OR = 3.80 | *P =* 0.004 |  |  |  |  |
|  |  | OR = 2.27 | *P* = 0.261 |  |  |  |  |
| S602I | TT | 272 (80.2) / 42 | 120 (61.9) | 12 (6.7) / 1 | 14 (12.7) | 272 (72.5) / 40 | 61 (53.5) |
| AGC→ATC | TG | 63 (18.6) / 11 | 61 (31.4) | 61(33.9) / 4 | 33 (30.0) | 83 (22.2) / 10 | 39 (34.2) |
|  | GG | 4 (1.2) / 0 | 13 (6.7) | 107 (59.4) / 15 | 63 (57.3) | 20 (5.3) / 5 | 14 (12.3) |
| EPTB | TT vs TG+GG | OR = 2.50 | *P* < 0.001 | OR = 0.49 | *P* = 0.080 | **OR = 2.29** | *P* < 0.001 |
|  |  | OR = 2.35 | *P* = 0.018 |  |  | **OR = 2.32** | *P* = 0.017 |
| **Continued** |  |  |  |  |  |  |  |
| ***TLR 4*** |  |  |  |  |  |  |  |
| D299G | AA | 281 (82.9) | 157 (80.9) | 159 (88.3) | 95 (86.4) | 353 (94.1) | 106 (92.9) |
| GAT→GGT | AG | 57 (16.8) | 36 (18.6) | 20 (11.1) | 14 (12.7) | 22 (5.9) | 8 (7.1) |
|  | GG | 1 (0.3) | 1 (0.5) | 1 (0.6) | 1 (0.9) | 0 (0) | 0 (0) |
|  | GG+AG vs AA | OR = 0.88 | *P* = 0.569 | 0.84 | *P* = 0.622 | 0.83 | *P* = 0.654 |
| T399I | CC | 325 | 178 | 161 (89.4) | 97 (88.2) | 357 | 108 |
| ACC→ATC | CT | 14 | 16 | 18 (10.0) | 12 (10.9) | 18 | 6 |
|  | TT | 0 | 0 | 1 (0.6) | 1 (0.9) | 0 | 0 |
|  | TT+CT vs CC |  |  | OR = 0.88 | *P* = 0.739 | OR = 0.91 | *P* = 0.841 |
| E474K | GG | 308 (90.9) / 49 | 184 (94.8) | 180 | 109 | 372 | 114 |
| GAA→AAA | GA | 30 (8.8) / 4 | 10 (5.2) | 0 | 1 | 3 | 0 |
|  | AA | 1 (0.3) / 0 | 0 (0) | 0 | 0 | 0 | 0 |
| EPTB | AA+GA vs GG | OR = 1.85 | *P* = 0.096 |  |  |  |  |
|  |  | OR = 1.50 | *P* = 0.504 |  |  |  |  |
| ***TLR 6*** |  |  |  |  |  |  |  |
| I120T | TT | 283 (83.5) / 45 | 176 (90.7) | 179 | 108 | 372 | 114 |
| ATT→ACT | TC | 52 (15.3) / 8 | 18 (9.3) | 1 | 2 | 3 | 0 |
|  | CC | 4 (1.2) / 0 | 0 (0) | 0 | 0 | 0 | 0 |
| EPTB | CC+TC vs TT | OR = 1.94 | *P* = 0.019 |  |  |  |  |
|  |  | OR = 1.74 | *P* = 0.221 |  |  |  |  |
| L194P | TT | 317 (93.5) / 50 | 174 (89.7) |  |  | 374 | 114 |
| CTG→CCG | TC | 22 (6.5) / 3 | 20 (10.3) |  |  | 1 | 0 |
| EPTB | TC vs TT | OR = 0.60 | *P* = 0.115 |  |  |  |  |
|  |  | OR = 0.52 | *P* = 0.302 |  |  |  |  |
| S249P | CC | 289 (85.2) / 44 | 137 (70.6) | 61 (33.9) / 3 | 38 (34.6) | 291 (77.6) / 43 | 78 (68.4) |
| TCA→CCA | TC | 47 (13.9) / 9 | 50 (25.8) | 88 (48.9) / 15 | 46 (41.8) | 72 (19.7) / 9 | 31 (27.2) |
|  | TT | 3 (0.9) / 0 | 7 (3.6) | 31 (17.2) / 2 | 26 (23.6) | 10 (2.7) / 3 | 5 (4.4) |
| EPTB | CC vs TC+TT | OR = 2.40 | P < 0.001 | OR = 0.97 | *P* = 0.909 | OR = 1.64 | *P* = 0.036 |
|  |  | OR = 2.03 | P = 0.071 | OR = 0.33 | *P* = 0.084 | OR = 1.65 | *P* = 0.187 |
| V427A | TT | 333 | 191 | 175 | 107 | 317 (84.5) / 46 | 111 (97.4) |
| GTT→GCT | TC | 6 | 3 | 5 | 3 | 53 (14.2) / 7 | 3 (2.6) |
|  | CC | 0 | 0 | 0 | 0 | 5 (1.3) / 2 | 0 (0) |
| EPTB | CC+TC vs TT |  |  |  |  | **OR = 6.77** | *P* < 0.001 |
|  |  |  |  |  |  | **OR = 7.24** | P = 0.001 |
| V465I | GG | 278 (82.0) / 42 | 179 (92.3) | 179 | 110 | 373 | 113 |
| GTT→ATT | GA | 59 (17.4) / 11 | 15 (6.7) | 1 | 0 | 2 | 1 |
|  | AA | 2 (0.6) / 0 | 0 (0) | 0 | 0 | 0 | 0 |
| EPTB | AA+GA vs GG | **OR = 2.62** | *P* = 0.001 |  |  |  |  |
|  |  | **OR = 3.13** | *P* = 0.006 |  |  |  |  |
| A474T | GG | 290 (85.5) / 42 | 180 (92.8) | 178 | 108 | 371 | 108 |
| GCT→ACT | GA | 46 (13.6) / 11 | 13 (6.7) | 2 | 2 | 4 | 6 |
|  | AA | 3 (0.9) / 0 | 1 (0.5) | 0 | 0 | 0 | 0 |
| EPTB | AA+GA vs GG | **OR = 2.17** | *P* = 0.013 |  |  |  |  |
|  |  | **OR = 3.63** | *P* = 0.002 |  |  |  |  |
| ***TLR10*** |  |  |  |  |  |  |  |
| A163S | GG | 313 (92.3) / 53 | 182 (93.8) | 163 (90.6) / 19 | 106 (96.4) | 267 (71.2) / 45 | 97 (85.1) |
| GCT→TCT | GT | 26 (7.7) / 0 | 12 (6.2) | 16 (8.9) / 1 | 4 (3.6) | 101 (26.9) / 9 | 17 (14.9) |
|  | TT | 0 (0) / 0 | 0 (0) | 1 (0.5) / 0 | 0 (0) | 7 (1.8) / 1 | 0 (0) |
| EPTB | TT+GT vs GG | OR = 1.25 | *P* = 0.522 | OR = 2.76 | *P* = 0.064 | OR = 2.31 | *P* = 0.003 |
|  |  |  |  |  |  | OR = 1.27 | P = 0.587 |
| N241H | AA | 79 (23.3)/ 12 | 47 (24.2) | 78 (43.3) / 10 | 52 (47.3) | 150 (40.0) / 28 | 61 (53.5) |
| AAT→CAT | AC | 172 (50.7)/ 27 | 100 (51.5) | 85 (47.2) / 7 | 52 (47.3) | 182 (48.5) / 23 | 41 (36.0) |
|  | CC | 88 (26.0) / 14 | 47 (24.3) | 17 (9.5) / 3 | 6 (5.4) | 43 (11.5) / 4 | 12 (10.5) |
| EPTB | CC+AC vs AA | OR = 1.05 | *P =* 0.809 | OR = 1.17 | *P* = 0.512 | OR = 1.73 | *P =* 0.011 |
|  |  | OR = 1.09 | P = 0.810 | OR = 0.90 | *P* = 0.822 | OR = 1.11 | *P* = 0.751 |
| V298A | GG | 317 (93.5) / 53 | 183 (94.3) | 162 (90.6) / 19 | 106 (96.4) | 275 (73.3) / 45 | 97 (85.1) |
| GTA→ATA | GA | 22 (6.5) / 0 | 11 (5.7) | 17 (9.4) / 1 | 4 (3.6) | 94 (25.1) /9 | 17 (14.9) |
|  | AA | 0 (0) / 0 | 0 (0) | 1 (0.5) | 0 (0) | 6 (1.6) / 1 | 0 (0) |
| EPTB | AA+GA vs GG | OR = 1.15 | *P =* 0.706 | OR = 2.94 | *P* = 0.047 | OR = 2.07 | *P =* 0.009 |
|  |  |  |  |  |  | OR = 1.27 | *P* = 0.587 |
| M326T | TT | 325 | 186 | 163 (90.6) | 106 (96.4) | 273 (72.8) / 46 | 96 (84.2) |
| ATG→ACG | TC | 14 | 8 | 16 (8.9) | 4 (3.6) | 96 (25.6) / 8 | 18 (15.8) |
|  | CC | 0 | 0 | 1 (0.5) | 0 (0) | 6 (1.6) / 1 | 0 (0) |
| EPTB | CC+TC vs TT |  |  | OR = 2.76 | *P* = 0.064 | OR = 1.99 | *P =* 0.013 |
|  |  |  |  |  |  | OR = 1.04 | *P* = 0.924 |
| I369L | AA | 72 (21.2) / 13 | 43 (22.2) | 76 (42.2) / 10 | 58 (52.7) | 148 (39.5) / 27 | 62 (54.4) |
| ATC→CTC | AC | 179 (52.8) / 28 | 103 (53.1) | 89 (49.4) / 8 | 46 (41.8) | 185 (49.3) / 24 | 42 (36.8) |
|  | CC | 88 (26.0) / 12 | 48 (24.7) | 15 (8.4) / 2 | 6 (5.5) | 42 (11.2) / 4 | 10 (8.8) |
| EPTB | CC+AC vs AA | OR = 1.06 | *P* = 0.803 | OR = 1.53 | *P* = 0.082 | OR = 1.83 | *P* = 0.005 |
|  |  | OR = 0.88 | *P* = 0.716 | OR = 1.22 | *P* = 0.822 | OR = 1.24 | *P* = 0.518 |
| I775V | AA | 320 (94.4) / 49 | 167 (86.1) | 138 (76.7) / 17 | 85 (77.3) | 298 (79.5) / 46 | 93 (81.6) |
| ATT→GTT | AG | 18 (5.3) / 4 | 24 (12.4) | 38 (21.1) / 3 | 22 (20) | 73 (19.5) / 9 | 20 (17.5) |
|  | GG | 1 (0.3) / 0 | 3 (1.5) | 4 (2.2) / 0 | 3 (2.7) | 4 (1.1) / 0 | 1 (0.9) |
| EPTB | GG+AG vs AA | OR = 2.72 | *P* = 0.001 | OR = 0.97 | *P* = 0.905 | OR = 0.87 | *P* = 0.622 |
|  |  | OR = 0.50 | P = 0.215 | OR = 0.68 | P = 0.566 | OR = 0.87 | P = 0.743 |

^*^ Allele frequency 0.03 was used as a cutoff line for rare and common variants in each ethnic group including cases and controls.

Odds ratio and P value are based on the comparison of certain genotypes of each common variant between cases and controls in specific ethnic group.

^†^ No. (%) of subjects

^#^ No. after slash indicates cases with extrapulmonary TB disease.

* ORs and P values were calculated for the comparison between extrapulmonary TB (EPTB) and TLR variants.
