# Supplementary material for: Full-Exon Resequencing Reveals Toll-Like Receptor Variants Contribute to Human Susceptibility to Tuberculosis Disease
Source: PLoS One. 2007 Dec 19;2(12):e1318. doi: 10.1371/journal.pone.0001318 (PMC2117342; doi:10.1371/journal.pone.0001318)
Supplement: Table S4 — Oligonucleotide primers used to amplify and sequence TLR1, 2, 4, 6, and 10 (0.06 MB PDF) [file pone.0001318.s005.pdf]

Table S4. Oligonucleotide primers used to amplify and sequence *TLR1, 2, 4, 6, and 10*

Genes	Primer name	Primer sequence (5'—3')
TLR1	TLR1-F1	AATGTCAGCCATGACTAATT
	TLR1-R1	CACAGTAGGGTGGCAAGAAATC
	TLR1-F2	TCAACCAGGAATTGGAATAC
	TLR1-R2	AGTTCCAGATTTGCTACAGT
	TLR1-F3	CATTTTATTTTGGATGTGTC
	TLR1-R3	TTCAAAAACCGTGTCTGTTA
	TLR1-F4	CCGTTCCTGCATTTGGATTT
	TLR1-R4	CTGGCAGCTCTGGAAGAAAT
	TLR1-F5	GCTTTAGCAGCCTTTCTGTA
	TLR1-R5	GTGCCCACTATATGAAATAA
	TLR1-F6	AGGAACATACCCTTAGAAGA
	TLR1-R6	CTTGCTCTGTCAGCTTAATA
	TLR1-F7	TCACAAGCTCAAAAGTCTCA
	TLR1-R7	AGTCACGGCATTTTCCAATA
TLR2	TLR2-F3	GGATGGTTGTGCTTTTAAGTACTG
	TLR2-F9	CTGTAAAAAGCCTTGACCTG
	TLR2-R12	GAACCAGGAAGACGATAAATTAG
	TLR2-F5	CCTGAGGAACTTGAGATTGATG
	TLR2-F15	TTTTCTGGTTCCTTGTTTACTTT
	TLR2-F13	CAGTGAAATTTGATGGTTGAAG
	TLR2-F7	ATCCAGCACACGAATACACAG
	TLR2-R10	GAGGGAGGCATCTGGTAGAG
	TLR2-F17	TACCTGTGTGACTCTCCATCC
	TLR2-F11	CTGTGGTATATGAAAATGATGTG
	TLR2-R6	AAGATCCCAACTAGACAAAGACTG

Supplementary Table 4 (continued)

Genes	Primer name	Primer sequence (5'—3')
TLR4	TLR4-F1	TTCTTCTAACTTCCTCTCCTGTG
	TLR4-R2	TTAGCTGTTCGGCTCTACTATGG
	TLR4-F3	TGCTCATCTTCTCTGTATCCTTC
	TLR4-R4	TACCCAGGACCATACCCAGGA
	TLR4-F5	TCTGTCCTGCTTGATGTCTTTG
	TLR4-R6	TGCCCACCTGGAAGACTCTG
	TLR4-F11	TAGAACAACTAGAACATCTG
	TLR4-R12	AGCTCCACCTGCTGCCTGAG
	TLR4-R14	TCAATAGTCACACTCACCAG
	TLR4-R10	CATTGAAAGCAACTCTGGTG
	TLR4-R8	AGATGAGACTCCACAAACCAAG
	TLR4-F13	CATCACATCTGTATGAAGAG
	TLR4-F7	GGTGTCCCAGCACTTCATCCAG
TLR6	TLR6-F1	ACAACCCTTTAGGATAGCCACTG
	TLR6-R4	AAACTCACAATAGGATGGCAGG
	TLR6-F5	ACCCTTCACCTTGTTTTTCAC
	TLR6-R6	CAGGCATTTCCAAGTCGTTTC
	TLR6-R8	CTTTAACTGACCTTCCTGGATG
	TLR6-F7	CCCCTGCTTTTATTGACCTCAT
	TLR6-R10	GAAACTTTGTCCCTGGCAAGAG
	TLR6-F9	GAGATTGTGATGGGCAAAATAG
	TLR6-R2	TAACCTCACCGCCTAGCTCAG
	TLR6-R12	TCTCTAACTGGCAGGCTAAC
TLR10	TLR10-F1	GTCGAAGACCCAATATACAG
	TLR10-R1	ATTAAGCAATAGAACCGATG
	TLR10-F2	CATCGGTTCTATTGCTTAAT
	TLR10-R2	CAGAGATGGGCTGAGAATGAAG
	TLR10-F3	CAGGTGCTTGCCCAAAAGTATT
	TLR10-R3	TTCCAGGAGAGCTTTCAGTT
	TLR10-F4	TTTGCCCACCACAATCTCTT
	TLR10-R4	TTCCATAAGCCCTTATAAAG
