# Supplementary material for: Full-Exon Resequencing Reveals Toll-Like Receptor Variants Contribute to Human Susceptibility to Tuberculosis Disease
Source: PLoS One. 2007 Dec 19;2(12):e1318. doi: 10.1371/journal.pone.0001318 (PMC2117342; doi:10.1371/journal.pone.0001318)
Supplement: Table S3 — Haplotype analysis of TLR6 genetic variants (0.04 MB PDF) [file pone.0001318.s004.pdf]

## Table S3. Haplotype analysis of *TLR6* genetic variants

| Haplotype | D30N | I120T | H125L | M156V | L194P | F209F | N227N | R247K | S249P | L251end | T255I | N257D | T287T | L298L | V327M | H345Y | T361T | L357L | G388E | T399M | L421L | V427A | H458R | N460del | V465I | A474T | Y567C | L572L | G592V | A644A | N690T | T756M | I777V |
|---|---|---|---|---|---|---|---|---|---|---|---|---|---|---|---|---|---|---|---|---|---|---|---|---|---|---|---|---|---|---|---|---|---|
| h0 | G | T | A | A | T | C | T | G | C | T | C | A | A | T | G | C | C | T | G | C | A | T | A | X | G | G | A | A | G | T | A | C | A |
| h1 | - | C | - | - | - | - | - | - | - | - | - | - | - | - | - | - | G | - | - | - | G | - | - | - | - | - | - | - | - | - | - | - | - |
| h2 | - | - | - | - | - | - | - | - | - | - | - | - | - | - | - | - | G | - | - | - | G | - | - | - | - | - | - | - | - | - | - | - | - |
| h3 | - | C | - | - | - | - | - | - | - | - | - | - | - | - | A | - | G | - | - | - | G | - | - | - | - | - | - | - | - | - | - | - | - |
| h4 | - | - | - | - | - | - | - | - | T | - | - | - | - | - | - | - | - | - | - | - | - | - | - | - | - | - | - | - | - | - | - | - | - |
| h5 | - | - | - | - | - | - | - | - | - | - | - | - | - | - | - | - | - | - | - | - | - | - | - | - | - | A | - | - | - | - | - | - | - |
| h6 | - | C | - | - | - | - | - | - | - | - | - | - | - | - | - | - | G | - | - | - | G | - | - | - | A | | - | - | - | - | - | - | - |
| h7 | - | - | - | - | C | - | - | - | - | - | - | - | G | - | - | - | G | - | - | - | G | - | - | - | - | - | - | - | - | - | - | - | - |
| h8 | - | - | - | - | - | - | - | - | - | - | - | - | - | - | - | - | G | - | - | - | G | - | - | - | A | - | - | - | - | - | - | - | - |
| h9 | - | - | - | - | - | - | - | - | - | - | - | - | - | - | - | - | G | - | - | - | G | - | - | - | - | - | - | - | - | G | - | - | - |
| h10 | - | - | - | - | C | - | - | - | - | - | - | - | G | - | - | - | - | - | - | - | G | - | - | - | - | - | - | - | - | - | - | - | - |
| h11 | A | - | - | - | - | - | - | - | - | - | - | - | - | - | - | - | G | - | - | - | G | - | - | - | A | - | - | - | - | - | - | - | - |
| h12 | - | - | - | G | C | - | - | - | - | - | - | - | G | - | - | - | - | - | - | - | G | - | - | - | - | - | - | - | - | - | - | - | - |
| h13 | - | - | - | - | - | - | C | - | - | - | - | - | - | - | - | - | G | - | - | - | G | - | - | - | - | - | - | - | - | - | - | - | - |
| h14 | - | - | - | - | - | - | - | A | - | - | - | - | - | - | - | - | G | - | - | - | G | - | - | - | - | - | - | - | - | - | - | - | - |
| h15 | - | - | - | - | - | - | - | - | - | A | - | - | - | - | - | - | G | - | - | - | G | - | - | - | - | - | - | - | - | - | - | - | - |
| h16 | - | - | - | - | - | - | - | - | - | - | T | - | - | - | - | - | G | - | - | - | G | - | - | - | - | - | - | - | - | - | - | - | - |
| h17 | - | - | - | - | - | - | - | - | - | - | - | - | - | - | - | - | - | - | - | - | - | C | - | - | - | - | - | - | - | - | - | - | - |
| h18 | - | - | - | - | - | - | - | - | - | - | - | - | - | - | - | - | - | - | - | - | - | - | G | - | - | - | - | - | - | - | - | - | - |
| h19 | - | C | - | - | - | - | - | - | - | - | - | - | - | - | - | - | G | - | - | - | G | - | - | X | - | - | - | - | - | - | - | - | - |
| h20 | - | - | - | - | - | - | - | - | - | - | - | - | - | - | - | - | G | - | - | - | G | - | - | - | - | - | - | G | - | G | - | - | - |
| h21 | - | - | - | - | - | - | - | - | - | - | - | - | - | - | - | - | G | - | - | - | G | - | - | - | - | - | - | - | - | - | C | - | - |
| h22 | - | - | - | - | - | - | - | - | - | - | - | - | - | - | - | - | G | - | - | - | G | - | - | - | - | - | - | - | - | - | - | T | - |
| h23 | - | - | T | - | - | - | - | - | - | - | - | - | - | - | - | - | G | - | - | - | G | - | - | - | - | - | - | - | - | G | - | - | - |
| h24 | - | - | - | - | - | - | - | - | - | - | - | G | - | - | - | - | G | - | - | - | G | - | - | - | - | - | - | - | - | - | - | - | - |
| h25 | - | - | - | - | - | - | - | - | - | - | - | - | - | - | - | T | - | C | - | - | - | C | - | - | - | - | - | - | - | - | - | - | - |
| h26 | - | - | - | - | - | - | - | - | - | - | - | - | - | - | - | - | - | - | A | - | - | - | - | - | - | - | - | - | - | - | - | - | - |
| h27 | - | - | - | - | - | - | - | - | - | - | - | - | - | - | - | - | G | - | - | T | G | - | - | - | - | - | - | - | - | - | - | - | - |
| h28 | - | - | - | - | - | - | - | - | - | - | - | - | - | - | - | - | G | - | - | - | G | - | - | - | - | - | G | - | - | - | - | - | - |
| h29 | - | - | - | - | - | - | - | - | - | - | - | - | - | - | - | - | G | - | - | - | G | - | - | - | - | - | - | - | T | - | - | - | - |
| h30 | - | - | - | - | - | - | - | - | - | - | - | - | - | - | - | - | G | - | - | - | G | - | - | - | - | - | - | - | - | - | - | - | G |
| h31 | - | - | - | - | - | - | - | - | - | - | - | - | - | C | - | - | G | - | - | - | G | - | - | - | - | - | - | - | - | - | - | - | - |
| h32 | - | - | - | - | - | T | - | - | - | - | - | - | - | - | - | - | - | - | - | - | - | - | - | - | - | - | - | - | - | - | - | - | - |
